# Recollections of England. 1841

**Published:** 1843-01

**Authors:** 


### ART. II.

*Erinnerungen an England.* 1841. Von Dr. MARX, Königl. Hannoverschem Hofrathe und Professor.—*Braunschweig*, 1842. 8vo, pp. 302.
*Recollections of England.* 1841. By Dr. K. F. H. MARX.

IT will be remembered that about a year and a half ago Professor Marx, of Göttingen, made himself known to many members of the medical profession in England, but particularly in London, as an ardent admirer of our country and its institutions, and as one not merely ready, but anxious to put the most favorable construction upon everything he saw and heard. The impressions made on his mind during his inspection of our country and its contents, were communicated from time to time to his brother, who is professor of natural philosophy at Brunswick. Being a physician he himself took the greatest interest in matters pertaining to medicine, and these consequently occupy the greater portion of the work. It is to these that our attention is necessarily and exclusively directed. We would state, however, that as Dr. Marx's brother was a layman, matters laical are also referred to and examined with the eye of a scholar, a gentleman, and of a studied but secluded philosopher. Of these matters laical we have only to say that all Dr. Marx has written is more or less amusing.

Our traveller made no stay in London after he had touched our shores, but went direct to Scotland, viâ York. His first breathing-time appears to have been in the modern Athens. As the impressions made upon his mind on his arrival there indicate much of the character of the author, we are induced to present his account of them:

"He whose inclination and duty it is to follow scientific pursuits can only breathe freely or feel at home where the higher educational establishments are found. To enjoy genuine satisfaction he must live in an intellectual atmosphere, and have objects presented to his notice interesting to his peculiar feelings. Nay, the very buildings around him must be congenial with his own pursuits. A visit to a fortress can scarcely be too short, although everything one sees is new and interesting. A mercantile town, with its bustle and hurry, its harbour and exchange, its warehouses and promenades, soon excites a desolate feeling of weariness. Even a metropolis, the residence of the court, with its parades, theatres, and saloons, affords but a transient excitement to the mind. It is quite otherwise in a place dedicated to the muses. Here the soul experiences an impressive tranquillity, an affectionate yearning, a sort of confidence and pride. It is like returning to our native home; like the enjoyment of the religion, the language, and the pursuits familiar to our hearts. Here one can understand how like seeks like. Here you may have that honorable position allotted to you, unrecognised by the world at large, and enjoy the peace and satisfaction only to be obtained in the society of intellectual men. Such were my reflections as I greeted the Penates of the Scottish university."

The museum of human and comparative anatomy in Surgeons' Hall, and of pathology, belonging to the university, are all noticed admiringly; as also Sir G. Ballingall, Sir C. Bell, Drs. Abercrombie, Christison, and Graham. Dr. John Thomson had a sort of pilgrimage made to him:

"As for many years I had set a great value on the works of Dr. John Thomson, I felt anxious to know him personally and to express my gratitude for his important researches. I heard with regret that he resided out of town, was ill, and had withdrawn himself from society. The last circumstance raised my curiosity, for I felt certain such a man would not abandon social life without some sufficient reason. I mentioned my wish to see him, at the hospital where his eldest son is professor of pathology. He expressed himself ready to accompany me to his father's; and one of the physicians present having placed his carriage at our disposal, we set out. We found the old man in bed; but I had scarcely had time to glance through the summer-house before he came to me, supported on a stick, and greeted me with affectionate cordiality. He took me by the hand, led me into the garden, thanked me for my visit. He spoke with so much animation, and such an unusual brilliancy appeared in his eye, and such tension (?—spannung) in his sunken features, that I began to feel anxious for him, and begged him to return. He seemed to suffer in his mind because many eminent men, with whom he was formerly on the best terms, had criticised his scientific opinions in a harsh and unguarded manner. He mentioned particularly his views respecting the treatment of syphilis without mercury. He felt that he was misapprehended, and with this impression ceased to publish; although this may have been rather in consequence of bodily weakness. He leaves to his sons (the younger of whom, the professor of physiology, was present,) his valuable collections, that through these justice may be done to him. I fully sympathised with him, and could only regret that I was under the necessity of declining his invitation to prolong my visit." (p. 25.)

The estimate of Dr. Abercrombie occupies nearly a page:

"John Abercrombie is one of the most popular physicians in Edinburgh. His published works exhibit accuracy of observation and soundness of judgment. He appears to be simple and unassuming in his manners; and that he is so in reality was the general opinion, so far as I could learn. It is generally understood that he goes too far in religious matters; and I remember to have heard and read the same opinion about him in Germany. The religious views of an individual belong exclusively to himself; and it always appeared to me an impertinence to make them the subject of common conversation. Certainly a piety which displays no trace of weakness of mind, of ostentation, or of hypocrisy ought not to be interfered with. Since I have discovered how much true religion is found in the family circles of this country, how much attendance at church is the result of feeling, and how generally the observances of religion are maintained as a duty—the exception only being remarkable—I should consider it hazardous to estimate an individual's character and qualifications from such circumstances. Certainly worthy persons may here be met with in the medical profession who have found religion to be their greatest solace under bereavement." (p. 26.)

And then we have a curious anecdote about Dr. Gooch.

Our professor made his way to the Highlands, and found the gymnastic exercises of his younger days to come in very usefully in facilitating his jumps from stone to stone among the bogs. He seems to have been delighted with the scenery, as we find him turning out for a walk at Callander before cockcrow: an early hour indeed, as the month was July.

Glasgow did not much interest our traveller. He left it without a sigh, and makes his way back to London with all the haste of a stray Cockney. "Paris," he says, "may be France, but London is England, and all the world beside." He visits diligently the metropolitan raree-shows, under the usual cicerones. The Zoological Gardens in Regent's Park are much, and not too highly esteemed:

"I now turned my steps towards Regent's Park, to see the Zoological Gardens. I have seen them—seen them by the hour, and shall see them again. When I hear that this institution, like similar ones in this country, is supported by private individuals, I cannot help asking how kings and emperors can bear comparison with these untitled folk. The park at Schönbrunn contains some beautiful and rare specimens, and the naturalist may be entertained by a ramble through the Jardin des Plantes, and will laugh heartily at the *Palais des Singes;* but here he feels himself as if in Paradise itself, when Eve gave names to all animals; or in Noah's ark, where of all creatures there was a pair. The representatives of every clime are here; and also not only of every genus, but even of species and varieties. I now saw, for the first time, a living orang-utang, and a giraffe with its young. A rhinoceros and a gigantic elephant attracted the young people: ladies rode on a little elephant. All but dangerous animals wander freely about in their inclosures, and thus afford an excellent opportunity for observing their habits." (p. 45.)

The professor compares the flow of population through the streets of London, and the traffic of ships and boats on the river to the circulation, as observed under the microscope, when the blood-globules are observed rolling after each other, over and on, in continuous succession. The Thames is the aorta of the world, and the Bank of England is the left ventricle of London—in many matters he might have said of the world too. Meditating upon the Thames Tunnel, it struck him that if Jupiter was so hard upon Prometheus for stealing fire, what would Neptune (supposing he had been alive) have done to Brunel for opening a road into his territories?

After a few days' residence in London our traveller forms acquaintances and begins to criticise persons as well as things:

"This morning I was at the British Museum, in the afternoon at the Hunterian, in the evening at Sir James Clark's—three things equally remarkable in their way. You are aware, from reading, how astonishingly rich the British Museum is in books, specimens of natural history, and works of art. I also had read a great deal about it; but who can describe a diamond? We must see the water, the brilliancy and the colour of it, for ourselves. The antediluvian animals and the Grecian monuments demonstrate the existence of bygone eras in creation and art. Forms so wonderful as the former no longer exist on earth, and the hand of man will never again strike that magic chisel which brought out the flowing drapery in the statues of some Athenian females.............. The museum of John Hunter is the pantheon of its founder; the monument of a life full of industry and research: a most instructive museum of comparative anatomy. Whoever has a taste for the study of this branch of science will see the whole of it before him. All structures, organs, and organized systems are there, arranged with reference to their functions and their regular development, from the lowest animals to the highest. The organization and final causes of animated structures are there exhibited to his astonished mind. The nature-worship here celebrated is lawful; for it is the admiration of creative power and human effort. At Clark's I learned to love English honesty and cordiality. The image of this excellent man is become part of myself." (p. 51.)

Our astonished and admiring traveller gets up next morning early to write again about what he had seen during the previous day, for he had been almost overwhelmed by his own thoughts. He adds, respecting the museum at the College of Surgeons, that it is a striking proof of what an individual may do, even with limited means, if he place his object clearly before him, and seek to attain it with prudence and perseverance. He alludes to the catalogues, and says;

"William Clift, the conservator, is a living catalogue. He is extremely kind and courteous, and having been educated under John Hunter, reveres his memory very much.......... Whoever has a recommendation to him, or is a zealous student may go at all hours and study practical physiology; if not so recommended the visits must be during the appointed hours. Among the curiosities are the skeleton of a dwarf, aged seven years, whose mother was delivered of it during a storm, and while enclasped by an ape; and the embalmed body of the wife of Van Butchell, the quack. Her relatives agreed to pay this man £500 per annum so long as she remained *above ground.* When she died he stowed her away carefully in a glass case, placed her in a room, and continued to draw the annuity. I must refer to the library at the college rather to notice the librarian, than the books. This office is held by Dr. Robert Willis, who, when young, made a pedestrian excursion through Germany, with knapsack on back, and knows German literature and physicians. He is a man of fine feelings, thoughtful, and truth-loving." (p. 55.)

A just tribute to Sir James Clark's worth is followed by a critique laudatory of the editor of the British and Foreign Medical Review, for which we tender our most respectful acknowledgments. Then come Sir B. Brodie, Mr. Travers, and a couple of phrenologists. Bright, Latham, Liston, Keate, and Lawrence are also visited.

"Although they all [all except the phrenologists] left on my mind an impression of sterlingness of character and frankness united with affability and kindness, I felt myself most attracted by Brodie and the elder Travers. Brodie is rather short and slenderly built, has a compressed mouth and winning intellectual eyes. He says little, but what he does say, has so much precision, and is so complete and comprehensive, that one cannot but wish to examine him more minutely, and in conversation, to follow up the experience and opinions of such an individual to their complete development. Travers is tall and strongly built, and active in spite of his age; true-hearted and social; clear in expressions as well as in judgement."

The two phrenologists give occasion for a hit at the "pseudo-Æsculapian sect."

"As I went to Guy's Hospital I was induced by their signs and handbills to go to two phrenologists. For the paltry sum of two guineas Mr. Donovan engages to teach you the difficult and useful art of learning people's interior from their exterior. That he was up to the thing he proved by feeling my head, and erected my horoscope with a diagnosis which dispelled all doubt about the matter. And truly his statements ought to have convinced me, as well as the opinion he gave respecting the individuals whose busts he had collected round him, and who, I believed, I knew either by their deeds or writings. Only there are innate aversions as well as ideas. Mr. Deville, near Exeter Hall, was out; nevertheless I went through his large collection of casts and skulls which is behind a lamp-warehouse. The doctrines transplanted to England by our countrymen, Gall and Spurzheim, afford a favorite pursuit to a portion of the higher classes; and have also given rise to a flourishing business. It may therefore easily happen that the bust of Elliotson, who is trying hard to bring animal magnetism into repute, should find little favour with the adherents of this pseudo-Æsculapian sect." (p. 57.)

Since our author's visit, however, phrenology and animal magnetism have shaken hands and kissed each other; and there is every promise of a goodly harvest under the conjunction of the star of the nativity-casters with that of the seers of the invisible.

As a companion-piece to the preceding, we give sketches of other pseudo-Æsculapians:

"A tooth-drawer recommends himself to the public by the comforting words 'toothach cured' displayed in his window; the letters being in the cuneiform character, and made up of carious teeth. A worm-doctor promises you the speediest possible cure; and modestly to insinuate that he may be depended on, close to his signboard 'Late Dr. Gardener's museum' is a collection of human and brute worms arranged in long bottles one above the other. The announcement 'dissenter medical office' made me curious to know what other remedies were used than those of 'high-church' medicine: they were Morison's pills."(p.58.)

Professor Marx thinks quackery on the decline in England, and thus accounts for the alleged fact:

"The herd of notorious quacks is much lessened, and also the number of nostrums. This circumstance, that is to say, the protection of the health against its most deadly foes, is rather to be attributed to the extension of right views among the lower orders, by means of cheap publications, than to the efforts of physicians or of a medical police. In fact the latter does not exist. It is the easiest thing imaginable to purchase poison; you may get it at the next shop; and either accidental or wilful cases of poisoning are occurring every day.

"You cannot go far in the streets without meeting a pock-marked person. We have forgotten in Germany that such a thing can happen, it is so rare. A man must travel into the native land of Jenner to witness an instance: and in this respect, he certainly will not become the panegyrist of unlimited personal freedom. In spite of the excellent institutions founded for the extension and encouragement of vaccination, and in spite of the numerous opportunities afforded for its being done gratuitously, many still inoculate for the smallpox, and raise a cry against vaccination—that truly God-like gift from medicine to man. Compulsory vaccination is not attempted, and could not easily be introduced or enforced." (p. 77.)

Following our traveller from St. George's in his journeyings eastward, we find him visiting St. Bartholomew's, Guy's, and St. Thomas' Hospitals; and from these he returns to University College. The museum at Guy's, Mr. Town's wax models, and Dr. Hodgkin's catalogue are noticed as being exceedingly meritorious. Surprise is also expressed at the extent of the London museums, especially of King's and University Colleges; and B. Cooper's, Langstaff's, and Kiernan's anatomical and pathological collections are referred to.

"Dr. Hodgkin is forty years old, but looks quite young; gentleness and kindness beam through every feature. Although in practice, he applies himself closely to pathological anatomy; and the collection at Guy's Hospital is almost his own creation; his works, as an author, are extremely good; and when we consider his multifarious engagements, it is matter of surprise how he can devote any portion of time to scientific pursuits." (p. 75.)

At University College the dissecting-room, the museum, and Dr. Carswell's drawings excited particular attention. The professor of comparative anatomy was found in his museum.

"Dr. Robert Grant is a modest Scotchman; following science quietly, and devoting his whole time to his own pursuits. He is a physician but does not practise, or rather, must not; since he refuses to comply with the strange regulations of the College of Physicians, which require him to pay handsomely for the permission to practise, although he got that permission long since at Edinburgh. He was acquainted with Cuvier and wrote his life in the Foreign (Quarterly) Review for 1830; Cuvier, while living, having communicated to him some particulars for this purpose." (p. 79.)

Dr. Paris carried the wandering professor off to two or three clubs, and raised admiring envy in his German heart as the only individual he ever met in the profession who had got a thousand pounds by a medical publication. A club, our comfort-loving bachelor (the professor *must* be a bachelor,) characterizes most poetically as an "Alpine height of pure air, a free port of leisure, a triumphal arch of comfort." To bachelors clubs are paradise itself; as everything is to be met with in them except wives and children. And then he gives a list of delights, ending with the cellar and kitchen, and shrewdly remarking that the latter, although prolific of every luxury, by no means emaciates the purse.

After the clubs comes the Isle of Wight. Beautiful he acknowledges it to be, but London is all in all to him; and back he flies to the witty, learned, and intellectual society of the metropolis. Then we have peeps into everything. The Penitentiary, the Duke of Wellington going to Parliament, Bedlam, drawing-room etiquette, (Dr. Marx is an impassioned admirer of our dear, fair, countrywomen,) carpets, stoves, and tobacco-smoking. The latter practice, curiously enough, is an abomination in his nostrils; and he gives it a "counterblaste." He quotes all the physicians he has met with, as authorities against the practice of smoking, and Dr. Prout especially, as maintaining that it changes the blood, so that it becomes of a yellowish or greenish colour; and this explains why smokers have such dirty cachectic complexions! The admirers of the hateful weed suffer also from stomach complaints, of which, and of diseases of the liver they often die. "But what's the use of talking?" says the professor. "The snuff-taker thinks it an agreeable thing to powder his nose and everybody about him with his artificial pollen; and the smoker considers that people ought to be much obliged to him for smoking into books, so that it is hateful to read in them after him." *De gustibus*, &c. We know and have heard of wise men and worthy men, aye noble men and royal too, who love a pipe, and have no stomach complaints, nor are likely to have or to die of diseased liver; although, at the same time, we altogether sympathise with Dr. Marx in his reprobation of the filthy custom. Amongst other things we must pass over our traveller's criticism on Mr. Wakley and eulogy of Dr. Holland, to make room for a visit to Hanwell and its "palace-like asylum." The day found convenient for the visit was a Sunday.

"Nine hundred and twelve madmen in one day! I may really be supposed to have spent a *madman's* Sunday. Yet I have seen this day much wisdom and philanthropy. Dr. Forbes took me to Hanwell Asylum, where Dr. Conolly, his friend, is the superintending physician. The air was warm; the sky a little clouded; a slight rain had laid the dust. In a while, the sun broke out, and his rays were reflected from myriads of glittering drops. The villages on the road were like sweet little towns; a great number of villas, all inviting from their neatness, elegance, and simplicity, alternated with parks and fertile fields. The numerous church-goers, the stage-coaches full of neatly-dressed young women, and the 'gigs' containing a single party, gave the eye occupation enough while the ear drunk in the chatty conversation of the friends, as the bee, the honey of flowers.

"The large establishment is in a pleasant situation, surrounded by beautiful gardens and quite detached. Unfortunately, Conolly was ill; so that his charming son and daughter were our guides. The latter, still of tender age, yet a vigorous young lady, wandered through the apartments of both the male and female lunatics like the good genius of the house. The noisy she stilled by a gentle reproof, the obstinate by a soothing appeal, the suffering by a comforting shake by the hand. Her father's principle lives in her, namely to treat the patients with the most perfect quietness of manner, and not to reason or talk much with them; as so doing only excites them. The person who is thoroughly firm and self-collected, while he exhibits the purest humanity, will certainly make the deepest and most beneficial impression. Here is practically shown man's power over man, when wielded with humanity. [We cannot resist the temptation of giving this last sentence in the original, as no translation can do justice to its idiomatic force and beauty: it ought to be engraved on the front of Hanwell, and over the name of JOHN CONOLLY:—*Hier wird durch die That bewiesen, was der Mensch über den Menschen durch das Menschliche vermag.*]

"The principal object of Dr. Conolly and the assistant-physicians is to be as friendly as possible with the patients, and win their confidence. This object is less readily attained by an imperious demeanour than by kindly expressions and a kindly manner. The assistants and keepers amount to eighty individuals. The matron, keepers, and nurses also exhibit a systematic feeling of kindness and affection towards the unfortunate inmates. Corporeal punishment is never practised, nor is a strait-waistcoat or girdle ever used. Here the use of these things is considered to be like adding fuel to fire. Watchfulness over the patients is the most important duty; but such measures as may assist and minister to the comfort of the keepers are not overlooked. The revolving chair is now never used. The means of restraint commonly practised—not as a punishment, but with the object of curing the patient or of maintaining order—is a seclusion for two or three hours in an airy court, or in an empty but clean apartment, so as even to remove the very idea of this being a measure of punishment. If the patient refuses to go in, he is not forced, but the other patients are sent away, and he is surrounded by courageous yet kind-hearted keepers who attempt to persuade him to go of his own accord. Force is only met by force, when the patient uses violence, and then only in self-defence, as a matter of necessity, and without appearance of passion.

"To remove wakefulness the patient is allowed to drink freely of cold water. When the shower bath is used to allay excitement, it is customary to place the patient in warm water up to above the middle; then to dry him quickly, cover him up warm, and send him to bed. Woollen clothing is considered the most suitable on account of the great variations in the weather; and also, because the insane have generally cold hands and feet. During the hot days of summer, a thinner clothing is permitted.

"A beautiful principle runs through the whole of this excellent physician's curative method, namely, still to consider himself as a learner, and the patients, whether well or ill-behaved, as the teachers from whom the right method is sure to be gleaned at last. Marks of attention and encouragement, instead of neglect, are often most useful in (apparently) incurable cases. The whole

4292

manner of individuals, who were all but given up on account of their intractable violence, is quickly changed when they are got to busy themselves with some decent and congenial occupation. Every patient is employed as much as possible; in the kitchen, washhouse, garden, or at handicraft occupations. Many lunatics being brought into the asylum quite uneducated, even unable to read, measures are taken to educate them. Several opportunities have been afforded for proving that the very first impression made on patients coming in, has produced a beneficial alteration in their state. Not a few have left this retreat quite well two or three months after their admission.

"According to the experience at Hanwell, the propensity to suicide is nearly always connected with some bodily disorder; and it is found to be the best plan to set the patients free from all restraint; simply watching them and keeping everything dangerous out of their way. The more obviously their actions are restricted, the more their mind becomes fixed on the propensity. Severity and restraint tend to keep the propensity alive. An attempt at suicide is frequently made by young girls, and is merely an hysterical symptom; on this account, everything should be kept as quiet as possible. In these cases, the best treatment is to moderate the too-busy proceedings of the bystanders, to send them about their business, and merely to keep the light excluded, the hands and feet cool with cold water, and all means of destruction carefully removed. ........ Going from the kitchen to the dining-rooms, you would scarcely believe that those who are so busy cutting potatoes, washing pots, carrying water and plates of provisions, are the same who only a few days previously disturbed everybody about them, and were the most refractory of idlers. Of the scourge which formerly was so rife in these institutions there is now no more trace; and only the hand remains ready to resuscitate the apparently dead in intellect. Instead of the chains which formerly riveted the maniac to the block, there is simply the feeling that kindness must ever be the attendant on misfortune."......(p. 140.)

Oxford was visited by our admiring friend, and its "remark-worthy" things observed with all the enthusiasm of the man of literature and the student; his graver observations being interspersed with a little small talk. Radcliffe's lucky escape from an unpleasant matrimonial engagement is detailed. A peep into the academic theatre gives him occasion to observe that it was the place in which Blucher was created a doctor after he had defended his thesis at Waterloo. The portrait of another master of disputations, the Duke of Wellington, in his robes as Chancellor, graces the same building.

Professor Marx informs us, to our great satisfaction, that he found Dr. Buckland domiciled in Christ Church College as comfortably as if he were in Abraham's bosom; and thinks there are few such reverends as he is, considered as a geologist, mineralogist, and a man of good common sense; not to mention his enjoyment of a pleasant joke. Our author had published an interesting memoir of Blumenbach (a translation of it appeared last year in the Edinburgh Philosophical Journal) in which among other things we find it stated that that master in science was a bad writer, on account of having been obliged to write with the left hand from having suffered an injury in the right. But Blumenbach had been a diplomatist at Paris, and had there learnt the trick of *writing in his pocket* on a bit of stiff paper. So when people quizzed his writing, he never failed to acknowledge its defects, but also never failed to ask his critic if he could write in his pocket? Mrs. Buckland being a lover of German had read this memoir of Marx's, and took the opportunity of asking our travelling critic if *he* could write in his pocket? He candidly acknowledges his want of that accomplishment, and broadly asserts it is by no means common with the Teutonic race. The architectural grandeur and princely abodes which learning exhibits at Oxford and Cambridge are unequalled, our professor thinks. It is evidently the first wish of his heart that Göttingen should be like them. It is certainly more like them than any other university in the world. Surely this may be somewhat consoling to him.

It was the fair at Oxford, and our author (who lets nothing escape him) observes that Oxford gingerbread is tougher and more clammy than German. We think there is some mistake here; he must have got hold of brandy-snap. The beds he found to be very comfortable, and the more so, because he could indulge the whim of stretching across them; being an agreeable change from the longitudinal reclination to which the descendants of Hermann are confined when dormant in their *interpulvinary couches.*

Indulging in some reflections on the constitution of the English universities, Professor Marx observes that the German universities lose much by not maintaining a connexion with their students after their introduction into life; a connexion secured by the peculiar constitution of the English universities, and to which they owe much of their present grandeur, wealth, and influence. We recommend this hint to the friends of the University of London.

While on a visit to a sick friend at South Lambeth, Professor Marx got initiated into the way of getting on in London. A physician must be a boasting, ubiquitous, bustling, intriguing fellow to get on fast, or indeed at all.

"Quiet merit, unless very great, is scarcely estimated. But the man who talks about his cures, makes himself conspicuous everywhere, and in addition has cunning enough to carry out his schemes, is considered by the public to be a practical man. On the other hand, the modest, learned, and thoroughly educated physician, who has not the tact or inclination to push himself forward, or who remains at home and only leaves his study when need is, passes for a learned man. It is thought he knows more of what is written in books than of what is done in the world. I once mentioned in company the name of a physician whose skill at the bedside I had often witnessed with admiration; and all that could be said of him was, that he was a bookworm. I answered with the remark that his solid acquirements in science constituted only the groundwork of his professional skill. But I was told without ceremony that that was not the case; that nobody had heard of his cures, and that it was only practical readiness that raised a physician." (p. 193.)

One can easily imagine how such doctrines must have jarred the feelings of an educated gentleman and an accomplished physician. It is too common for half-educated quacking practitioners so to abuse the public ear, and utter such sentiments against not only an educated but a highly skilful practitioner. The plan has the double object of making their own ignorance meritorious, and the merits of their competitor injurious to his success. We hope that those who are seeking to restrain quackery will begin with quacks of this description, as the most unrighteous and dangerous of the whole tribe.

The character of the English physician, though sketched with the pen of an admirer, is, on the whole, given correctly. And first as regards his scientific acquirements:

"The English physicians know little of scientific medicine. They are wanting in systems. With them theory has not interpenetrated experience. Anything of the kind they display is no more than an insipid eclecticism. They think little of arrangements and deductions founded on or flowing from fundamental principles. Their science is not the product of pure thought; it is a mosaic work of observations. If subjects of the kind be brought under the notice of an Englishman, he scarcely understands you; and when you try to make him comprehend your meaning, he will say, we must be just before we are generous. They insist that people should busy themselves with substantial realities and not fine-sounding words. The construction of philosophical theories, and the dressing up and arrangement of facts and general propositions are certainly not their forte. But it would be matter for regret if they were to fritter away their sound sense, enlightened judgment, and innate truthfulness in an infinity of subtle distinctions; or become mystified by the opinions of the theorizing metaphysical school. But in fact, their native love of freedom repels the stays and strait-waistcoats of a system. Inhabitants of a country which carries on an intercourse with the whole world, they are accustomed to a wide horizon, and from an early period are practised to distinguish the apparent from the real. They think that this is peculiarly the duty of the physician; that he must examine each fact in its varied and almost infinite relations as clearly, accurately, and precisely as possible; and then arrange the results logically, expressing them under plain and perspicuous propositions. Poets only may be permitted to weave the silken threads of imagination, and philosophers plunge into the depths of pure thought to bring up mere idealities, useless fantasies and systems, or wander after cold and desolate generalities. I had already formed opinions of this kind respecting the English physicians and the tendency of their scientific pursuits from a perusal of their writings. A personal acquaintance with them at the bed-side, in their public institutions, and in their schools, has only served to fix and extend these opinions." (p. 204.)

A young practitioner pointed out two carriages at the door of a house, with the remark, that they belonged to two distinguished physicians, who, although not on good terms, had often to meet each other in consultations. This circumstance affords an opportunity for expressing an opinion on the moral character of the profession:

"The understanding between physicians is otherwise very good. Love and hate are everywhere; but especially in a country where ready money gives influence, and influence, honour; and where party-spirit finds herself at home. But they meet often in private company, particularly at table, and are often called into consultation together; so that an undisturbed and calm judgment is enabled to triumph over prejudices and feelings. The consequence of this good collegiate understanding among themselves, in connexion with their gentlemanly demeanour, their independent circumstances, their superior general education, their moral integrity, and their great skill in the practice of their profession, is, to be so honoured and esteemed by the public, that you can never hear a disparaging or injurious remark spoken against their profession." (p. 208.)

A call at a medical bookseller's gives our author occasion to remark on the evident want of a taste for the study of the valuable old authors. The bookseller, the young practitioner, and our author, are each made to express their sentiments in a dialogue, the first-mentioned concluding it with the assertion that a complete edition of the medical classics would be a bad speculation, and would only be undertaken by subscription. This bookseller further adds, that so far as he is acquainted with living medical authors he doubts whether the older writers have any attractions for them.

It is possible that Professor Marx has not rightly interpreted this bookseller's sentiments, but has supposed that the writing public is, in England, as numerous a body as in Germany, and guides the reading public. This is manifestly a mistake. We are ready to admit, that hitherto a taste for the older medical authors has not been generally diffused. That deficiency, however, has arisen simply from the general want of a good preliminary education. These old authors are the poesy, antiquities, and belles lettres of medicine, and it will be found that just as the standard of general education is raised in the profession the taste for them will be increased. This taste is in process of development; and, in proof, we need only refer to the wishes made public, from time to time, for the formation of a society that shall expressly devote itself to the republication of the medical classics, ancient and modern. Several gentlemen have, within these two years, taken pains to recommend such a society, and we understand it is now forming. We heartily wish it success; and the more heartily, because such a society would be able to republish the works of the authors alluded to in a cheap, and at the same time, neat form. The perusal of these is the proper finish to the mere academic education of the practitioner. They might be read during the leisure of commencing practice; and, while they would solace the many anxious hours peculiar to that period of life, would enable the practitioner to appreciate the merits of those literary charlatans who conceal a few old sterling truths in a mass of their own nonsense, and then, with incredible confidence of assertion, pass them off on the medical public as their own great discoveries.

Our traveller passes from medical literature to practice, and gives the reasons why, in his opinion, our doses of medicine and our treatment generally, are so Herculean. The patient is not visited sufficiently often to carry out the expectant plan; and the people are so accustomed to strong foods and drinks, that they have little reliance on any other than strong medicine. This, the forty-second letter, is concluded with some reflections upon medical authorship in England, which are interesting.

In the remaining fifteen letters we find numerous matters and things viewed, re-viewed, and meditated on;—Dr. Paris, and Sir J. South; Cambridge, and the peculiarities of English Universities; Broome Park, and its respected proprietor, Sir B. Brodie, of whom we have a just estimate; Mr. Guthrie, and a comical note about the great cost of becoming a surgical examiner; Dr. Holland, who, treating Trojan and Tyrian alike, had just come from a professional visit to Viscount Melbourne, and was just going on the same errand to the Earl of Aberdeen; University and King's Colleges; Dr. Sharpey and Thomas Morton; hospital practice and dispensaries. In the last letter, dated from Paris, we have traveller's gossip; a comparison of the two nations he had visited, by no means favorable to the French; and a sigh divided between England and Fatherland.

We had marked several passages for special notice, but our allotted space is filled. We can only say that the "Reminiscences," written as they were, on the spur of the moment, are extremely interesting from their graphic yet simple style. As our German friend has behaved so handsomely to our countrymen, while criticising them, we cannot do less than close our critique on himself with an adieu in the high German fashion, yet sincerely from the heart,—so we say to him,

